# Ultrastructural and Immunohistochemical Study of a Gastric Bizarre Leiomyoma: Bizarre Nuclei and Prominent Cytoplasmic Processes Extending Into and Displacing the Stroma

**DOI:** 10.1111/pin.70014

**Published:** 2025-04-14

**Authors:** Kaoru Furihata, Waka Iwashita, Atsushi Kurabayashi, Kojima Koji, Mutsuo Furihata

**Affiliations:** ^1^ Department of Pathology Kochi Medical School Kochi University Nankoku Kochi Japan; ^2^ Department of Gastroenterology Division of Internal Medicine, Tosa Municipal Hospital Tosa Kochi Japan

**Keywords:** electron microscope, immunohistochemistry, leiomyoma, stomach

## Abstract

This study presents the ultrastructural and immunohistochemical findings of a gastric bizarre leiomyoma arising in the vestibule of a 79‐year‐old male. Histologically, loosely proliferating tumor cells consist of large, multinucleated, bizarre nuclei with intranuclear inclusions and abundant cytoplasm‐containing vacuoles. A murky line was apparent between the tumor cells and eosinophilic and heterogenous stroma‐like areas. Immunohistochemically, tumor cells exhibited positively stained dot patterns of α‐smooth muscle actin and caldesmon, which were distributed in the cytoplasm of tumor cells and stroma‐like regions. Ultrastructurally, tumor cells exhibited extended and complex cytoplasmic processes comprising the fascicles of filamentous fibers. These structures were also detected in the apparent stroma‐like regions observed histologically and were consistent with the α‐smooth muscle actin‐ and caldesmon‐immunopositive dot structures. The original stromal areas remained as considerably narrow gaps between tumor cells with extended cytoplasmic processes. To the best of our knowledge, this is the first report detailing the unique ultrastructural and immunohistochemical characteristics of tumor cells and the limited stromal composition of an extremely rare primary gastric bizarre leiomyoma.

## Graphical abstract

In this report, we describe an extremely rare case of primary gastric bizarre leiomyoma that is histologically consistent with leiomyoma with bizarre nuclei in the uterus. Ultrastructurally, tumor cells had extended and complex cytoplasmic processes comprising the fascicles of filamentous fibers which were also detected in the apparent stroma‐like regions observed histologically and were consistent with the caldesmon‐immunopositive dot structures. We reveal the distinctive characteristics of tumor cells and the limited stromal composition of this neoplasm.

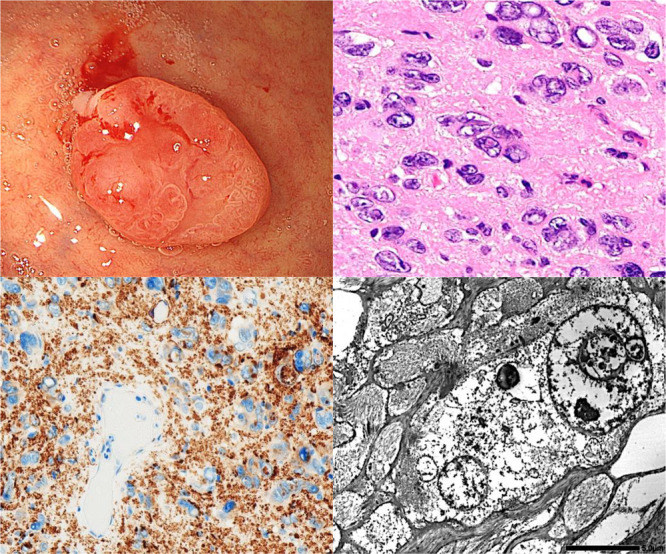

**Abbreviations**
BL: bizarre leiomyoma
GISTs: gastrointestinal stromal tumors
LBN: leiomyoma with bizarre nuclei
LMB: leiomyoblastoma
LMS: leiomyosarcoma
α‐SMA: α‐smooth muscle actin

## 1 Introduction

Leiomyoma with bizarre nuclei (LBN), characterized by the presence of bizarre nuclei with severe atypia and referred to as symplastic leiomyoma, represents a distinct pathological entity within the broader category of leiomyomas. In 1909, Kelly and Cullen first described LBN as a subtype of leiomyoma occurring in the uterus [1]. Following comprehensive investigations, LBN was defined as a leiomyoma variant characterized by the presence of bizarre cells as per the 2020 WHO classification [2]. Although LBN typically follows a benign clinical course [3, 4], a few cases (1.9%) have exhibited recurrence [5]. Severe nuclear atypia is a characteristic finding suggestive of leiomyosarcoma (LMS) [2, 6, 7], whereas some LBN cases may exhibit severe mitosis or necrosis [8]. Therefore, these two entities must be accurately distinguished [9] because their differential diagnosis has significant clinical implications. Leiomyoma is the most common benign mesenchymal neoplasm found in the stomach [10, 11]. Gastric LBN is a distinctive and exceedingly uncommon neoplasm. Our literature search yielded no case reports of primary gastric LBN.

This study describes a rare case of primary gastric bizarre leiomyoma (BL) that histologically conforms to LBN in the uterus. To the best of our knowledge, this is the first report of a patient with BL exhibiting the ultrastructural and immunohistochemical characteristics of tumor cells with prominent cytoplasmic processes and considerably narrow stromal areas. We discussed the distinctive characteristics of this case in comparison with other documented cases of smooth muscle tumors, particularly with reference to LBN.

## 2 Clinical Summary

**Figure 1:**
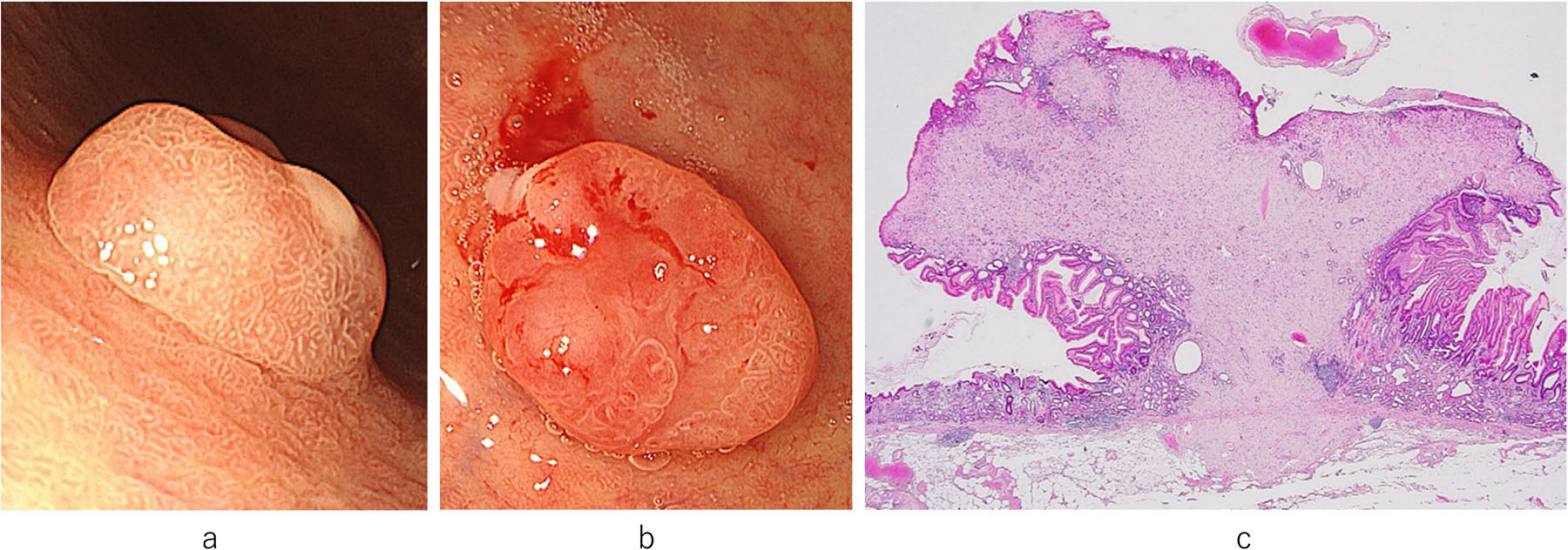
A polypoid lesion (10 mm diameter) in the greater curvature of the upper body of the stomach. (a) Lateral view showing the circumscribed polypoid lesion covered by mucosa. (b) Partial erosion at the upper surface of the lesion. (c) Gross examination reveals an externally protruding polypoid lesion in the stomach. Histologically, the circumscribed, unencapsulated tumor is located in the mucosa and focally extends into the submucosa.

A male patient in his 70s visited our hospital with a chief complaint of abdominal distention for several months. Endoscopy results revealed the presence of a protruding, polypoid lesion with partial surface ulceration in the greater curvature of the upper body of the stomach (Figure 1a,b), which was subsequently excised via endoscopic submucosal dissection.

## 3 Pathologic Findings

**Figure 2:**
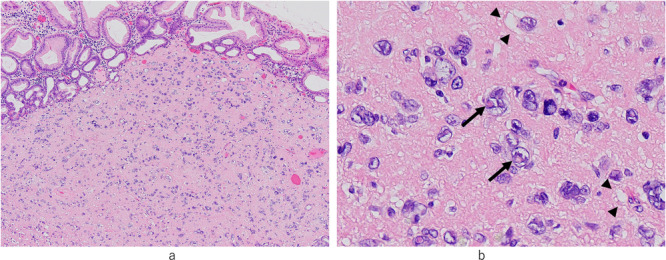
(a) Low‐magnification image showing a gastric LBN. Loosely proliferating tumor cells beneath the crypt are arranged individually or in partial clusters set against a background of eosinophilic stroma‐like material. (b) High magnification image showing tumor cells in gastric LBN with large, multinucleated, and bizarre nuclei. Intranuclear cytoplasmic inclusions and cytoplasmic vacuoles are also present in the tumor cells.

Gross examination revealed that the well‐circumscribed but unencapsulated protruding tumor, with a maximum diameter of 1.5 cm, was predominantly located in the mucosa and partially extended to the submucosa (Figure 1c). Submucosal arteriosclerosis with thrombosis and venous congestion were also noted. Histologically, tumor cells were arranged in loosely proliferating individual or intermingling aggregate patterns, set against a background of an eosinophilic and heterogeneous stroma‐like substance (Figure 2a). The tumor was composed of atypical cells featuring notably large, multinucleated, and bizarre nuclei, which showed distinct nucleoli and intranuclear cytoplasmic inclusions. Tumor cells also contained abundant cytoplasm‐containing vacuoles. A murky line was apparent between tumor cells and eosinophilic stroma‐like areas (Figure 2b). Mitotic figures, lymphatic or vascular invasion, or necrotic foci were not observed.

**Figure 3:**
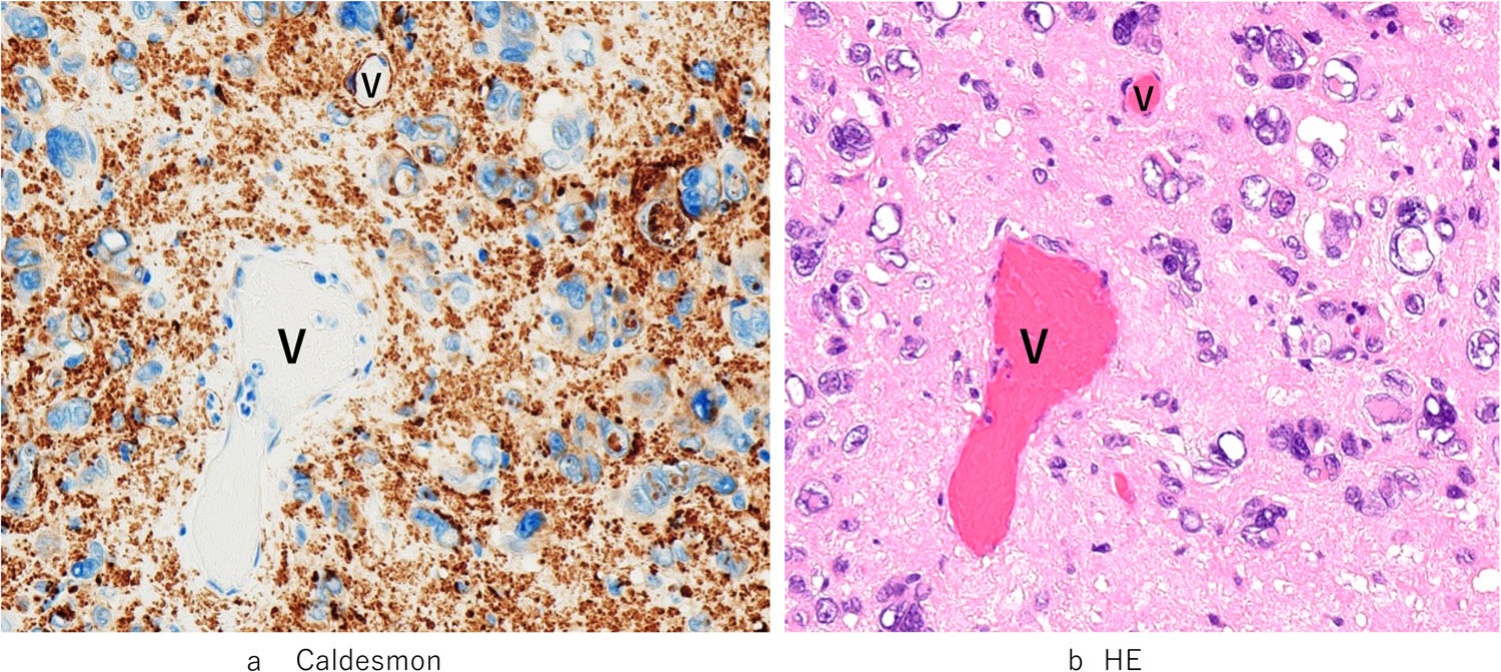
Two consecutive panels showing immunostaining images of a gastric BL: (a) immunostaining using the anti‐h‐caldesmon antibody and (b) hematoxylin and eosin staining. Tumor cells exhibit positively stained dot patterns following anti‐h‐caldesmon immunostaining. The cytoplasm of tumor cells exhibiting positively stained dot patterns upon anti‐h‐caldesmon immunostaining. The eosinophilic stroma‐like area surrounding tumor cells and vessels also display the same dot patterns as those observed upon caldesmon immunostaining.

Immunohistochemistry, using the streptavidin–biotin–peroxidase complex method (for antibodies and immunoreaction results, refer to Table 1), showed dot‐like α‐smooth muscle actin (α‐SMA) and caldesmon positivity in the cytoplasm of tumor cells, with positivity extending away from the nucleus. The unstructured eosinophilic stroma‐like components surrounding the tumor cells also exhibited immunopositivity for these two antibodies (Figure 3a). Focal, weak positivity for desmin was detected in the tumor cell cytoplasm. The Ki‐67 labeling index was under 1% in tumor cells, with no positive Ki‐67 immunostaining in cells with bizarre nuclei. As detailed in Table 1, no positivity was observed for p53, c‐kit, CD34, DOG1, S‐100, HMB45, MDM2, CDK4, ER, PgR, or AE1/3 antibodies.

**Table 1:** Used antibodies and immunohistochemical findings in tumor cells.

Antibodies	Tumor cells
α‐SMA	++ (Cytoplasm)
h‐Caldesmon	++ (Cytoplasm)
Desmin	+ (Cytoplasm)
Ki‐67	+ (Nucleus < 1%)
p53	—
c‐kit	—
CD34	—
DOG1	—
S‐100	—
HMB45	—
MDM2	—
CDK4	—
ER	—
PgR	—
AE1/3	—

*Note:* ++, diffuse strongly positive; +, focal weakly positive; −, negative.

**Figure 4:**
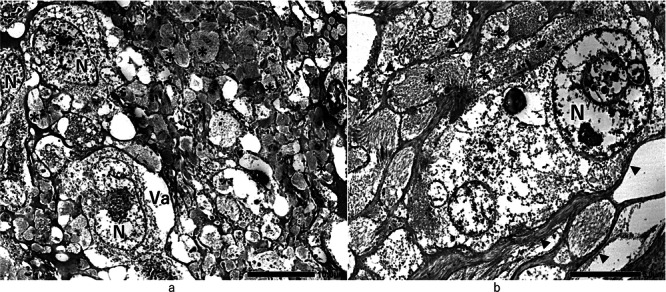
(a) Electron micrographs showing tumor cells with polymorphic nuclei (N) and cytoplasmic vacuoles (Va) surrounded by several irregularly round‐ to oval‐shaped vesicles containing fascicles of myofilamentous fibers (*). Vesicular structures are consistent with the distributions of caldesmon‐positive dot patterns detected in stroma‐like areas, as shown in Figure 3. (b) Vesicles comprising myofilamentous fibers form in succession within the tumor cytoplasm, indicating that the tumor cells have complex cytoplasmic extensions consisting of myofilamentous fibers (*). Original stroma appears as narrow gaps between tumor cells and their cytoplasmic processes (arrowheads).

For electron microscopic analysis, small fragments of the buffered formalin‐fixed tumor were further fixed using glutaraldehyde and OsO_4_, followed by embedding in epoxy resin. These results revealed tumor cells comprising large irregularly shaped nuclei with prominent nucleoli and abundant cytoplasm‐containing vacuoles around the nucleus (Figure 4a). Tumor cells were surrounded by several irregularly round‐to‐oval‐shaped vesicles comprising fascicles of myofilamentous fibers (Figure 4b). These vesicular structures were consistent with the distribution patterns of α‐SMA and caldesmon‐positive dot structures that were detected in the stroma‐like areas using immunohistochemical methods (Figure 3). These vesicles, comprising myofilamentous fibers, are formed in succession within the tumor cytoplasm. This indicates that tumor cells have complex cytoplasmic extensions containing myofilamentous fibers (Figure 4b). The original stroma was present as narrow gaps between tumor cells that had prominent cytoplasmic processes.

## 4 Discussion

LBN is a histologic leiomyoma subtype that typically occurs in the uterine corpus [2]. A medical literature review in PubMed using the keywords “leiomyoma bizarre” and “stomach” revealed that most cases exhibited characteristics like those observed in leiomyoblastoma (LMB), a diagnosis initially proposed by Stout in 1962 [12]. LMB was previously considered a distinct type of smooth muscle tumor, characterized by round to polygonal cells, frequently exhibiting vacuoles or clear regions around the nuclei and an epithelial‐like morphology differing from that of typical leiomyoma, which is composed of spindle cells [13]. Therefore, LBN must be carefully diagnosed and differentiated from LMS. Some LBNs display overlapping histopathologic features with LMS [14], which can have severe clinical consequences, particularly if LBN is misdiagnosed as LMS. The features of the gastric LBN‐like tumor presented in this study confirmed that most of the histological characteristics are consistent with those of LBN, such as large tumor cells with pleomorphic and multinucleated bizarre nuclei [2]. Additionally, the presence of large intranuclear inclusions is a hallmark of this histologic LBN subtype [15]. Immunopositivity for myogenic markers, such as α‐SMA and caldesmon, along with ultrastructural findings, for example, fascicles of myofilamentous fibers in the cytoplasm of tumor cells, supports the possibility of myogenic differentiation of this neoplasm [16]. These results indicate that this gastric LBN‐like tumor originates from smooth muscle tissue and reflects the pathology of LBN observed in gynecological cases [2].

Smooth muscle tumors, such as leiomyoma and LMS, have been reported in the gastrointestinal tract [10]. Gastrointestinal leiomyomas typically show minimal atypia with rare or no mitotic figures, whereas LMS exhibits marked atypia and enlarged, pleomorphic, polychromatic nuclei [10]. Pathological mitotic activity and necrosis are critical indicators of sarcomatous lesions, including gastric LMS [17]. Given the rarity of gastric LBN, awareness and accurate diagnosis of LMS and LBN are essential, as they have distinct therapeutic implications. Unlike gastric LMS, the gastric LBN‐like tumor in this case study did not show histological features suggesting malignancy, such as mitotic figures, vessel invasion, necrotic foci, or a high Ki‐67 labeling index. The features of the reported tumor support its benignity, aligning with those observed in uterine LBNs [18, 19].

The present LBN‐like tumor must also be differentiated from gastrointestinal stromal tumors (GISTs), characterized by c‐kit mutations [20]. As shown in Table 1, the lack of positivity for c‐kit, CD34, and DOG1 rules out GIST. Similarly, neural tumors, vascular tumors, solitary fibrous tumors, dedifferentiated liposarcomas, and PEComas can be excluded. Consequently, the neoplasm in this report was diagnosed as a gastric BL. Electron microscopy revealed the ultrastructure of tumor cells, particularly highlighting their extensive cytoplasm and complex, elongated cytoplasmic processes comprising fascicles of myofilamentous fibers. These processes also replaced the eosinophilic, heterogeneous stroma‐like structures identified histologically and corresponded to the α‐SMA‐ and caldesmon‐positive dot structures detected immunohistochemically. The remaining original stroma was sparsely distributed between tumor cells, which exhibited prominent cytoplasmic processes. Although the dynamic expansion of intricately expanding cytoplasmic processes of tumor cells requires further investigation, ultrastructural and immunohistochemical analyses are critical for elucidating the histological architecture of tumor cells and stromal composition in gastric BLs. To the best of our knowledge, this is the first report of an extremely rare type of primary gastric BL revealing the unique ultrastructural and immunohistochemical characteristics of large tumor cells and sparse stroma. Further evaluation of gastric BL cases, along with uterine LBNs and comprehensive assessments of multiple smooth muscle tumors, may provide more insight into the characteristics and histogenesis of this tumor.

## Author Contributions

**Kaoru Furihata:** writing – original draft, writing – review and editing. **Waka Iwashita:** visualization, writing – original draft. **Atsushi Kurabayashi:** visualization, writing – original draft. **Mutsuo Furihata:** writing – review and editing. **Kojima Koji:** writing – review and editing.

## Disclosure

The authors have nothing to report.

## Ethics Statement

Informed consent was obtained from all patients. This study was conducted in accordance with the Declaration of Helsinki 1975.

## Conflicts of Interest

The authors declare no conflicts of interest.
